# Current-Era Outcomes of Balloon Aortic Valvotomy in Neonates and Infants

**DOI:** 10.1016/j.jacadv.2022.100004

**Published:** 2022-03-16

**Authors:** Anders H. Christensen, Rajiv R. Chaturvedi, Connor P. Callahan, Kyong-Jin Lee, Andrea Wan, David J. Barron, Osami Honjo, Lee N. Benson

**Affiliations:** a Department of Pediatrics, The Labatt Family Heart Centre, Division of Cardiology, The Hospital for Sick Children, Temerty Faculty of Medicine, Toronto, Ontario, Canada; b Department of Pediatric Cardiology, Oslo University Hospital, Rikshospitalet, Oslo, Norway; c Division of Cardiovascular Surgery, The Hospital for Sick Children, Temerty Faculty of Medicine, Toronto, Ontario, Canada; d Stanford University School of Medicine, Palo Alto, California, USA

**Keywords:** aortic valve replacement, aortic valve stenosis, balloon aortic valvotomy, congenital heart disease, reintervention, surgical aortic valvotomy

## Abstract

**Background:**

The optimal initial treatment pathway for aortic valve stenosis remains debated.

**Objectives:**

The objective of this study was to review current outcomes of balloon aortic valvotomy (BAV) in neonates and infants.

**Methods:**

Neonates and infants with a biventricular circulation treated with BAV between 2004 and 2019 were reviewed.

**Results:**

One hundred thirty-nine infants (48% neonates) with median (Q1, Q3) age of 33(7, 84) days and weight 4.0 (3.4, 5.1) kg were followed up for 7.1 (3.3, 11.0) years. BAV reduced peak-to-peak gradient from mean (SD) 52 (16) mmHg to 18 (12) mmHg; *P* < 0.001. Aortic regurgitation (AI) increased with time after BAV. Three children died during follow-up. Fifty-one reinterventions (26 BAV, 19 aortic valve replacements [AVRs], and 6 surgical valvotomies) were performed on 40 children. Freedom from AVR (95% CI) was 96% (93%-99%) at 1, 91% (86%-96%) at 5, and 86% (79%-93%) at 10 years. The predictors of AVR were a unicommissural valve (hazard ratio [HR] [95% CI]: 3.7 [1.4-9.6]; *P* = 0.007) and moderate to severe AI after index BAV (HR [95% CI]: 3.3 [1.1-9.7]; *P* = 0.029). Freedom from reintervention was 84% (78%-90%) at 1, 76% (69%-83%) at 5, and 69% (60-78%) at 10 years. Main predictors of reintervention were age below 1 month (HR [95% CI]: 2.1 [1.1-4.1]; *P* = 0.032) and postdilation peak-to-peak gradient (per 10-mmHg increase; HR [95% CI]: 1.36 [1.02-1.79]; *P* = 0.032).

**Conclusions:**

BAV is a safe and effective treatment for aortic valve stenosis in neonates and infants. Outcomes are competitive with contemporary published data on aortic valve repair in relation to mortality, gradient relief, long-term AVR, and reintervention rates. In the absence of significant AI, surgery can be reserved for those with gradients resistant to valve dilation.

## Teaser

One hundred thirty-nine neonates and infants with a biventricular circulation treated with balloon aortic valvotomy (BAV) were reviewed. BAV reduced peak-to-peak gradient from mean (SD) 52 (16) to 18 (12) mmHg, *P* < 0.001. Median (Q1, Q3) follow-up was 7.1 (3.3, 11.0) years. Aortic regurgitation increased with time after index BAV. Freedom from aortic valve replacement (95% CI) was 96% (93-99%) at 1, 91% (86-96%) at 5, and 86% (79-93%) at 10 years. Freedom from reintervention was 84% (78-90%) at 1, 76% (69-83%) at 5, and 69% (60-78%) at 10 years. Contemporary experience with BAV shows it is a safe and effective treatment option in the youngest of children.

Aortic valve stenosis (AS) accounts for about 5% of congenital heart lesions.^1^ It is a lifelong condition often requiring multiple procedures. Surgical aortic valvotomy (SAV), first described by Lillehei et al^2^ in 1956, was the only available treatment option for almost 3 decades, until Lababidi et al^3^^,^^4^ published the technique of balloon aortic valvotomy (BAV) in 1983. Despite decades of procedural experience, the initial choice between surgery- or catheter-based treatment strategies remains controversial, particularly in the youngest of patients. In 2001, the Congenital Heart Surgeons Society study documented similar outcomes between SAV and BAV in neonates and infants,^5^ and as such, many centers adopted BAV as initial treatment.

While refinements in technique and low-profile balloons have allowed BAV to become safe and effective,^6^, ^7^, ^8^, ^9^, ^10^ surgery has also evolved from simple commissurotomy to valve reconstruction.^11^ Single-center comparative studies have reported improved longevity of stenosis relief, increased freedom from reintervention, and less aortic regurgitation (AI) in those treated with SAV.^11^, ^12^, ^13^ Others report that outcomes remain similar, even after matching for age, weight, and valve morphology.^14^ Comparing results between studies remain challenging, particularly due to the heterogeneity in the study populations.

This study reports contemporary outcomes of BAV performed on neonates and infants. Factors assessed include intermediate- and long-term safety, efficacy, and longevity of outflow tract gradient reduction, the degree of AI, reintervention and valve replacement rates, and factors predicting suboptimal outcomes.

## Methods

### Design and study population

This study is a retrospective review of children from birth to 1 year of age with congenital AS whose index procedure was a BAV at the Hospital for Sick Children, Toronto, Canada, between January 2004 and July 2019. BAV was the institutional treatment of choice for children meeting standard clinical criteria for intervention (ie, critical AS with ductal dependency and/or depressed left ventricular [LV] function, invasive peak-to-peak gradient >50 mmHg, nonsedated mean Doppler gradient >50 mmHg).^15^ SAV was reserved for children with additional lesions that required open heart surgery. Excluded were infants with 1) a non–apex-forming LV treated with a hybrid procedure or single ventricle palliation; 2) Shone complex physiology; 3) polyvalvar disease; and 4) primary pulmonary hypertension. Children meeting inclusion criteria were identified from the hospital and catheterization databases. Clinical information was deidentified according to institutional and governmental policies. The study protocol was approved by the Research Ethics Board (REB # 1000067899). Informed consent was obtained from all patients prior to the balloon aortic valvulotomy, but individual consent for study participation was not required.

### BAV and invasive hemodynamics

The catheterization procedures were performed using standard techniques^15^ under general anesthesia by 1 of 4 operators. Access site and the use of rapid right ventricular pacing for balloon stabilization depended on the child’s size, ventricular function, and operator preference. Aortic valve diameter was evaluated by angiography, and gradients measured by pull-back pressures. The degree of AI was assessed by echocardiography. Initial balloon diameters were 90-100% of the aortic valve annulus. Procedural complications assessed included 1) arterial thrombosis requiring long-term anticoagulation; 2) arrhythmias with hemodynamic consequences requiring intervention; 3) mitral regurgitation; and 4) the development and degree of AI.

### Echocardiography

Echocardiographic images were retrospectively analyzed by a nonblinded single reviewer (A.H.C.). For study participants followed up outside the institution, echocardiographic reports were obtained from their primary cardiologists. Echocardiographic data were collected at the following time points: 1) before the index BAV; 2) within 1-2 days after the index BAV; 3) prior to any reintervention; and 4) at the end of follow-up (at either aortic valve replacement [AVR] or the last clinic visit). AS was grouped by mean gradient as none to mild (0-20 mmHg), moderate (20-40 mmHg), or severe (>40 mmHg). AI was categorized as non/trivial, mild, moderate, or severe using standard qualitative and quantitative signs of valvar dysfunction.^16^ The aortic valve annulus was measured in parasternal long axis, LV dimensions, and ejection fractions were obtained from short-axis M-mode. Aortic valve morphology was categorized as unicommissural, bicommissural, or tricommissural from the predilation echocardiograms. Mitral stenosis was defined as a mean gradient >4 mmHg. Having multiple left-sided obstructions was defined as AS plus at least 2 of the following: mitral stenosis, LV outflow tract obstruction, and/or aortic arch obstruction.

### Statistics

Statistical analyses were carried out using SPSS, version 26 (IBM Corp) and R, version 4.0.3 (R Foundation for Statistical Computing). Continuous data are presented as mean ± SD or median with range or quartiles (Q1, Q3) depending on distribution. Categorical data are reported as frequencies, and cumulative survival is given as a percent with 95% confidence interval (CI). The fraction of missing data was <5% and not imputed. Statistical tests of differences were performed using Student t-test, Mann-Whitney U, Wilcoxon signed rank test, Chi square test, or Fishers exact test as appropriate. All tests were carried out two-sided. A probability value <0.05 was set as the level of statistical significance.

### Survival plots and hazard ratio analyses

Time-to-event analyses were performed with: 1) time to reintervention (SAV, BAV, or AVR); 2) time to repeat BAV; and 3) time to AVR. Differences between Kaplan-Maier subgroup curves were evaluated using log-rank testing. Univariate and multivariate Cox proportional hazard regression analyses were used to identify factors at index BAV affecting event-free survival. The following potential predictors were tested: sex, age <1 month, weight, symptomatic heart failure, ductal dependency, unicommissural aortic valve, ejection fraction before BAV, LV internal end-diastolic diameter before BAV, multiple left heart obstructions, moderate or severe AI after BAV, aortic invasive peak-to-peak gradient before and after BAV, mean echocardiographic gradient after BAV, use of rapid right ventricular pacing, diameter of the aortic valve annulus, and balloon-to-aortic valve annulus diameter ratio. Predictors not mentioned in the results section did not meet statistical significance. Predictors in the multivariate models were chosen on the basis of clinical relevance, avoiding highly correlated variables, with sample size considerations limiting the number of possible covariates due to the relative rare occurrence of events.

## Results

### Patient characteristics

Of 157 children treated with BAV in the study period, 10 were excluded with a non–apex-forming LV requiring a hybrid procedure or single ventricle palliation, 4 with severe Shone complex physiology, 3 with severe polyvalvar disease, and 1 with severe primary pulmonary hypertension (Supplemental Figure 1). One hundred thirty-nine children (71% males) with median age of 33 (range: 0-354) days and weight 4.0 (range: 1.1-9.5) kg were reviewed (Table 1). Thirty-two (23%) of the children had heart failure, and 35 (25%) were duct dependent at the index procedure. Associated lesions are described in Table 1.

**Table 1:** Demographics

Characteristics	
Male	98 (71%)
Age at inclusion, d	33 (7-84)
Age group at inclusion	
0-1 mo	66 (48%)
1-3 mo	40 (29%)
3-12 mo	33 (24%)
Weight at inclusion, kg	4.0 (3.4-5.1)
Associated lesions	
None	103 (74%)
Coarctation of the aorta	15 (11%)
Mitral stenosis	9 (7%)
Multiple left obstructions	5 (4%)
Interruption of the aortic arch	4 (3%)
Other	2 (1%)
Symptoms	
Asymptomatic	76 (55%)
Tachypnea	30 (22%)
Heart failure	32 (23%)
Duct dependent	35 (25%)
Aortic valve annulus, mm	7.5 (6.3-8.5)
Aortic valve morphology	
Unicommissural	29 (21%)
Bicommissural	106 (76%)
Tricommissural	4 (3%)
Timing of events	
Length of follow-up, years	7.1 (3.3-11.0)
Time to reintervention, years	
Balloon aortic valvotomy (n = 24)	0.25 (0.13-0.81)
Surgical aortic valvotomy (n = 6)	1.2 (0.01, 4.9)
AVR (Ross n = 17, prosthetic valve n = 2)^a^	3.7 (0.95, 7.7)

Values are n (%) or median (Q1-Q3).

Having multiple left obstructions was defined as aortic stenosis plus 2 of mitral stenosis, left outflow tract obstruction or aortic arch obstruction. Patients with Shone complex physiology were excluded.

AVR = aortic valve replacement.

a Time to prosthetic valve was 13.0 and 12.5 years.

### Index BAV

Access was obtained through the femoral artery in 118 (85%), internal carotid artery in 9 (7%), femoral vein in 10 (7%), and umbilical vein in 2 (1%) children. Balloon stabilization with rapid right ventricular pacing was used in 33 of 66 neonates (50%) and in 46 of 73 (63%) children >1 month of age. The maximal-balloon-diameter-to-aortic-valve annulus ratio was median 1.0 (0.92, 1.0). The index BAV acutely reduced the invasively measured peak-to-peak gradient from 52 (21) to 18 (11) mmHg, mean peak systolic Doppler gradient from 96 (30) to 49 (21) mmHg, and mean Doppler gradient from 53 (16) to 26 (12) mmHg (Supplemental Table 1). The number of children with mild AI increased from 2 (1%) to 56 (42%), moderate AI from 0 (0%) to 13 (10%), and severe AI from 0 (0%) to 5 (4%).

Eighty-seven (63%) children did not have any significant complication associated with the index procedure. Arterial thrombosis requiring up to 3 months of treatment with low-molecular-weight heparin occurred in 39 (28%) and was evenly distributed among neonates and infants. Seven children (5%) had hemodynamically significant arrhythmias requiring intervention. One infant had a cardiac arrest and was cannulated for extracorporeal membrane oxygenation but recovered after 18 minutes of conventional resuscitation. A second infant developed moderate mitral valve regurgitation after the use of a transvenous antegrade approach crossing the mitral valve with the balloon catheter. A third infant developed an intimal tear of the lesser curvature of the transverse arch related to a 0.014-inch wire exposed by a monorail coronary balloon; the child did not require additional interventions. A fourth child required femoral artery reconstruction after retrieval of the arterial sheath shaft after it separated from the sheath hub.

### Clinical course and reintervention and AVR rates

The median length of follow-up was 7.1 (3.3, 11.0) years. Three (2%) children died during follow-up, all remote from BAV (range: 4 months to 7 years) and unrelated to severe AI. All deaths occurred after surgical AVR. One death occurred in the postoperative period, while the other 2 were late and unrelated to the surgical procedure. There were no heart transplants. Peak and mean Doppler gradients did not change over the cause of the study, and the mean values were 46 (25) mmHg and 25 (14) mmHg at the last follow-up (Supplemental Table 1). The burden of AI increased with time. At the last follow-up, 31 (23%) had none or trivial, 43 (32%) mild, 34 (25%) moderate, and 27 (20%) had severe AI.

**Figure 1:**
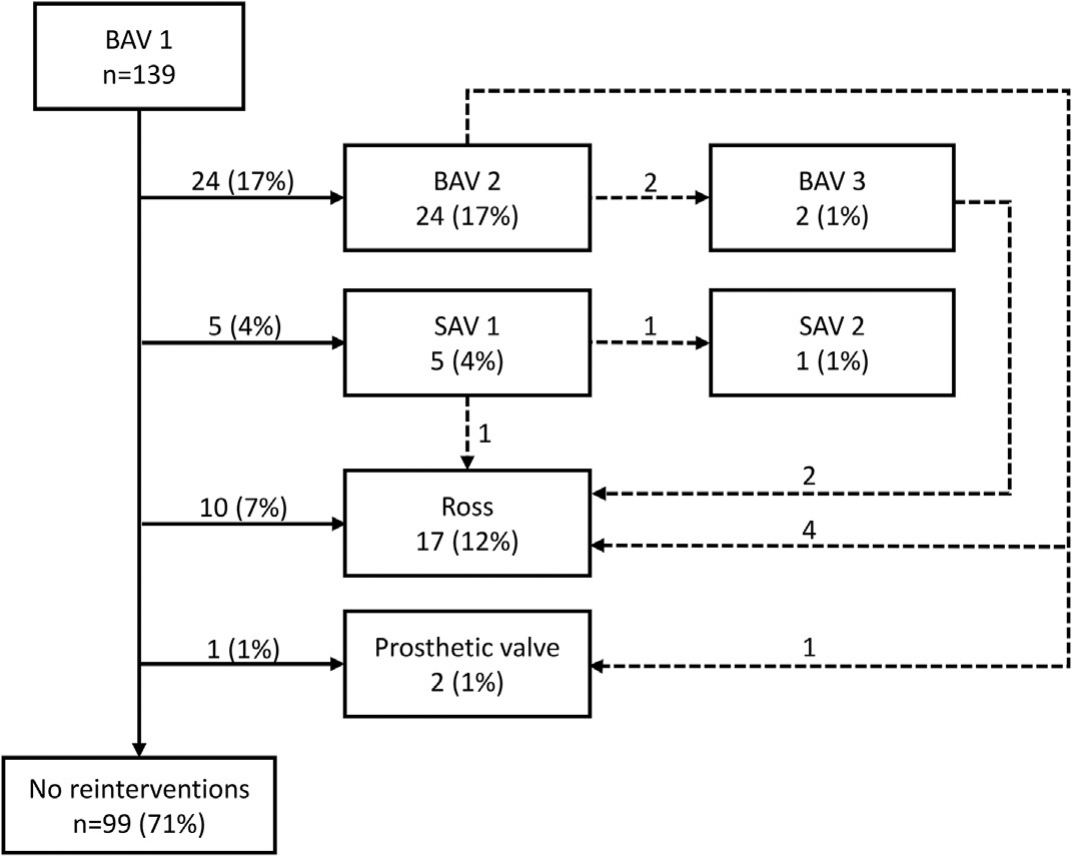
Patient Treatment Courses Flowchart describing patient treatment courses. A total of 190 procedures were performed on 139 children. BAV = balloon aortic valvotomy; SAV = surgical aortic valvotomy.

Ninety-nine (71%) children were reintervention free, and 40 (29%) underwent 51 reinterventions (Figure 1). Seven of 24 (29%) children who required a second BAV had an AVR, and 2 children underwent a Ross procedure after a third BAV. Indications for SAV were evenly distributed between AS (n = 2), AI (n = 2), and mixed lesions (n = 2). Indications for AVR were AS (n = 5), AI (n = 9), or mixed lesions (n = 7).

**Figure 2:**
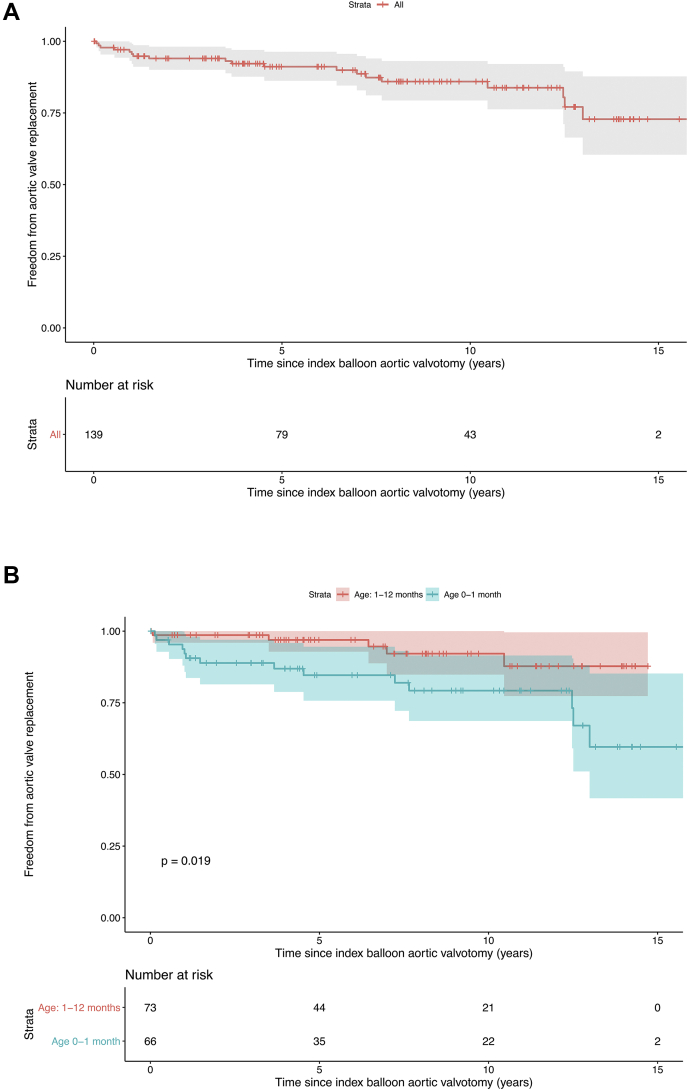

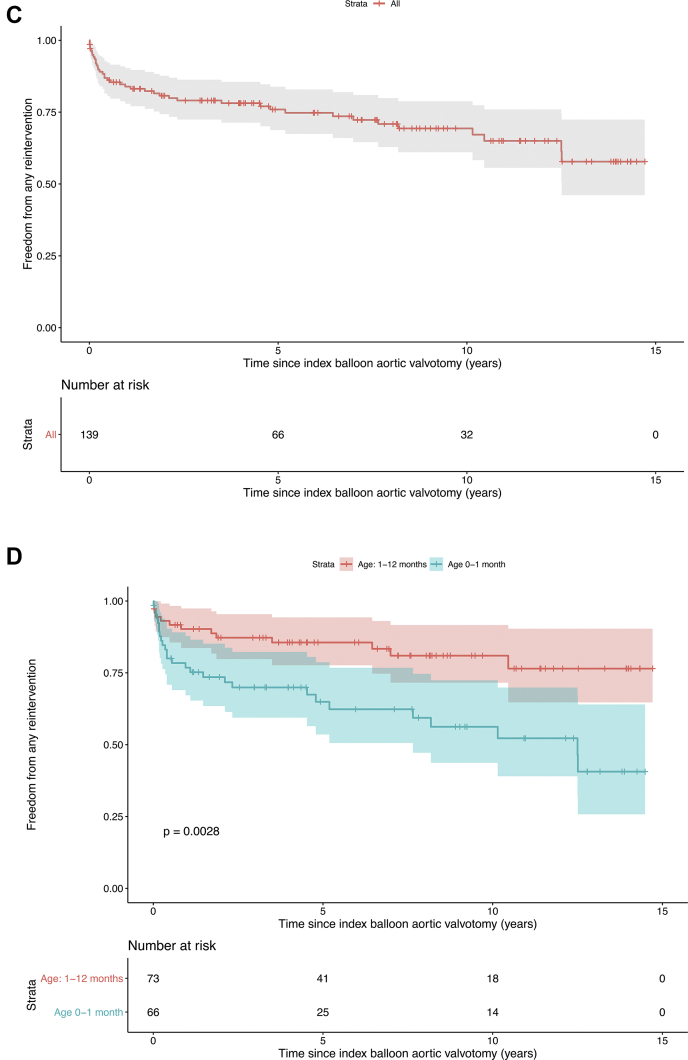
Cumulative Event-Free Survival Cumulative freedom from aortic valve replacement **(A and B)** and valve reintervention **(C and D)**. In **B and D**, the children were divided into subgroups according to age <1 month and age 1 to 12 months. Subgroup differences were examined with log-rank testing. Neonates have shorter event-free survival than infants.

Freedom from: 1) any reintervention was 84 ± 6% at 1 year, 76 ± 7% at 5 years, and 69 ± 9% at 10 years; 2) repeat BAV was 88 ± 5% at 1 year, 83 ± 6% at 5 years, and 82 ± 7% at 10 years; and 3) AVR was 96 ± 3% at 1 year, 91 ± 5% at 5 years, and 86 ± 7% at 10 years (Figure 2, Central Illustration). In children aged <1 month: 1) freedom from any reintervention was 77 ± 10% at 1 year, 65 ± 13% at 5 years, and 56 ± 14% at 10 years; and 2) freedom from AVR was 92 ± 7% at 1 year, 85 ± 9% at 5 years, and 79 ± 11% at 10 years.

**Central Illustration:**
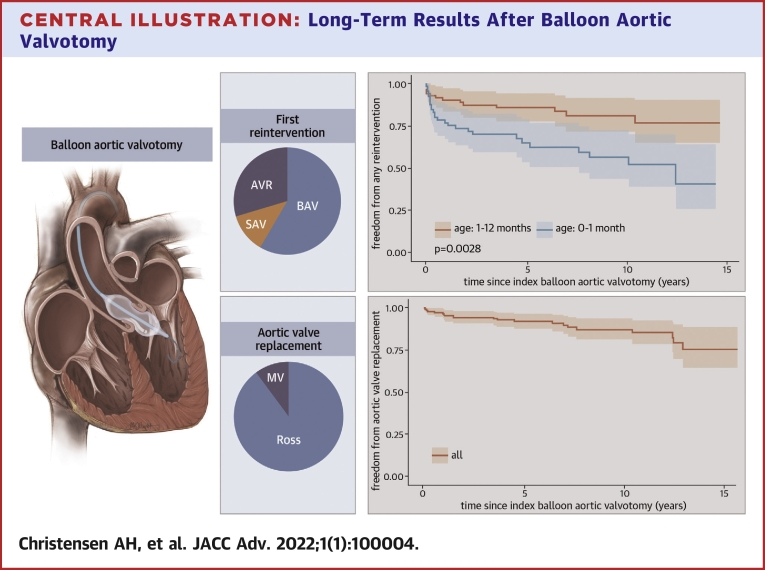
Long-Term Results After Balloon Aortic Valvotomy During balloon dilation the aortic stenosis is relieved by inflating a low-profile balloon positioned across the valve annulus. A visible “waist” on the balloon resolves as the valve is dilated. The most common first reintervention after BAV is a second BAV, while the majority of AVRs were done with the Ross procedure. Cumulative freedom from any reintervention was 69% at 10 years, with neonates having a shorter event-free survival. Cumulative freedom from AVR was 86% at 10 years. AVR = aortic valve replacement; BAV = balloon aortic valvotomy; MV = mechanical valve; SAV = surgical aortic valvotomy.

### Factors associated with reintervention and AVR

A subgroup analysis (Table 2) compared event-free survivors to those requiring a reintervention. The reintervention-free group was older, weighed more, had fewer males, had fewer children with heart failure or ductal dependency, had higher mean Doppler gradients (but equal invasively measured peak-to-peak gradient), and had higher LV ejection fractions before the index BAV. Doppler gradients fell more in response to the index BAV, and LV ejection fractions remained higher in the reintervention-free group, while the degrees of AS and AI were similar. Length of follow-up did not differ between the subgroups. Similar to the total population, reintervention-free survivors had on average unchanged Doppler gradients and a time-dependent increase in AI. An isolated analysis of neonates with and without reinterventions showed similar patterns and is presented in Table 3.

**Table 2:** Differences Between Children Requiring Reintervention and Event-Free Survivors. Long-Term Outcomes of Children With Event-Free Survival

	Any Reintervention (n = 40)	95% CI	Event Free (n = 99)	95% CI	*P* Value
Background					
Male	33 (83%)		65 (66%)		**0.049**
Age at iBAV, d	8 (4-8)	3-23	42 (11-106)	34-67	**<0.001**
Weight at iBAV, kg	4.0 (1.3)	3.6-4.4	4.7 (2.0)	4.4-5.1	**0.027**
Length of follow-up, y	7.1 ± 4.9	5.6-8.7	7.1 ± 4.4	6.3-8.0	0.985
Heart failure before iBAV	17 (43%)		15 (15%)		**<0.001**
Duct dependent	18 (45%)		17 (17%)		**<0.001**
Unicommissural valve	12 (30%)		17 (17%)		0.092
Catheterization					
AoV peak-to-peak gradient before iBAV, mmHg	51 ± 21	44-58	51 ± 21	47-56	0.889
AoV peak-to-peak gradient after iBAV, mmHg	20 ± 15	15-25	17 ± 10	15-19	0.148
Echocardiography					
AoV mean gradient before iBAV, mmHg	47 ± 20	41-54	56 ± 13	53-59	**0.003**
AoV mean gradient after iBAV, mmHg	28 ± 14	24-33	24 ± 11	22-26	0.061
AoV mean gradient reduction from iBAV, mmHg	19 ± 22	12-26	31 ± 17	27-34	**0.001**
Ejection fraction before iBAV, %	56 ± 22	49-64	71 ± 14	68-74	**0.001**
Ejection fraction after iBAV, %	63 ± 19	57-70	72 ± 12	69-75	**0.015**
AoV regurgitation after iBAV					
None or trivial	19 (48%)		40 (43%)		0.164
Mild	13 (33%)		43 (46%)		
Moderate	7 (18%)		6 (6%)		
Severe	1 (3%)		4 (4%)		
**Long-Term Outcomes in Event-Free Survivors**	**After iBAV**		**Final Visit**		
AoV peak Doppler gradient, mmHg	48 ± 20	43-52	44 ± 20	40-48	0.145
AoV mean Doppler gradient, mmHg	25 ± 11	23-28	23 ± 14	21-25	0.184
AoV stenosis					0.323
Non/mild (mean gradient <20 mmHg)	37 (41%)		38 (43%)		
Moderate (mean gradient 20-40 mmHg)	44 (48%)		44 (49%)		
Severe (mean gradient >40 mmHg)	10 (11%)		7 (8%)		
Aortic valve regurgitation					**<0.001**
None or trivial	40 (43%)		23 (24%)		
Mild	43 (46%)		35 (37%)		
Moderate	6 (6%)		20 (21%)		
Severe	4 (4%)		18 (19%)		

Values are n (%), median (Q1-Q3), or mean ± SD, unless otherwise indicated.

Statistical differences were examined using Student *t*-test, Mann-Whitney *U* test, Wilcoxon signed rank test, Chi square test, or Fishers exact test as appropriate. The final visit was median 7.1 years after iBAV. The **bold***P* values indicate significance at the 0.05 level.

AoV = aortic valve; CI = confidence interval; iBAV = index balloon aortic valvotomy.

**Table 3:** Differences Between Neonates Requiring Reintervention and Neonates With Event-Free Survival

	Any Reintervention (n = 27)	95% CI	Event Free (n = 39)	95% CI	*P* Value
Background					
Male	23 (85%)		29 (74%)		0.290
Age at iBAV, d	3 (3-7)	2-5	7 (3-18)	5-13	**0.005**
Weight at iBAV, kg	3.5 ± 0.5	3.2-3.7	3.3 ± 0.8	3.1-3.6	0.517
Length of follow-up, y	7.3 ± 5.1	5.3-9.4	6.6 ± 4.7	5.1-8.2	0.643
Heart failure before iBAV	12 (44%)		9 (23%)		**0.010**
Duct dependent	18 (67%)		14 (36%)		**0.014**
Unicommissural valve	11 (41%)		11 (28%)		0.426
Catheterization					
AoV peak-to-peak gradient before iBAV, mmHg	54 ± 21	45-62	62 ± 22	55-69	0.139
AoV peak-to-peak gradient after iBAV, mmHg	17 ± 11	13-22	19 ± 11	16-23	0.514
Echocardiography					
AoV mean gradient before iBAV, mmHg	45 ± 20	37-53	56 ± 15	51-61	**0.011**
AoV mean gradient after iBAV, mmHg	28 ± 12	23-32	24 ± 12	20-28	0.294
AoV mean gradient reduction from iBAV, mmHg	17 ± 19	10-25	31 ± 18	25-37	**0.003**
Ejection fraction before iBAV, %	53 ± 23	42-64	67 ± 13	63-72	**0.005**
Ejection fraction after iBAV, %	64 ± 19	56-72	73 ± 10	70-77	**0.024**
AoV regurgitation after iBAV					0.377
Non or trivial	11 (41%)		16 (42%)		
Mild	10 (37%)		18 (46%)		
Moderate	5 (19%)		2 (5%)		
Severe	1 (4%)		2 (5%)		

Values are n (%), median (Q1, Q3), or mean ± SD, unless otherwise indicated. Statistical differences were examined using Student *t*-test, Mann-Whitney *U* test, Chi square test, or Fishers exact test as appropriate. Neonates are children <1 month of age. The **bold***P* values indicate significance at the 0.05 level.

AoV = aortic valve; CI = confidence interval; iBAV = index balloon aortic valvotomy.

Univariate Cox regression analyses revealed that age <1 month, lower patient weight, smaller aortic valve annulus diameter, unicommissural aortic valve, symptoms of heart failure, ductal dependency, low LV ejection fraction before BAV, and a high residual aortic valve gradient increased, while use of right ventricular pacing decreased, the risk of reintervention (Table 4). Age <1 month, unicommissural aortic valve, heart failure, ductal dependency, and low ejection fraction before BAV increased the risk of AVR. Moderate or severe AI after the index BAV did not increase the hazard ratio for AVR in the univariate model but became significant when adjusting for aortic valve residual gradient and aortic valve morphology in the multivariate model. In further multivariate analyses, age <1 month and high residual aortic valve gradient predicted the need for any form of reintervention, while unicommissural aortic valve and high residual aortic valve gradient predicted the need for repeat BAV (Table 4).

**Table 4:** Factors at iBAV Influencing Need of Reintervention and AVR

	Any Reintervention		BAV Reintervention		AVR	
	HR (95% CI)	*P* Value	HR (95% CI)	*P* Value	HR (95% CI)	*P* Value
Univariate analyses						
Age 0-1 month at iBAV	2.65 (1.36-5.14)	**0.004**	2.94 (1.22-7.09)	**0.016**	3.19 (1.15-8.86)	**0.026**
Weight at iBAV (per 1-kg increase)	0.81 (0.66-0.99)	**0.037**	0.72 (0.54-0.96)	**0.014**	0.90 (0.68-1.18)	0.433
AoV annulus diameter at iBAV (per 1-mm increase)	0.71 (0.57-0.88)	**0.002**	0.52 (0.38-0.73)	**<0.001**	0.84 (0.63-1.13)	0.253
Unicommissural aortic valve	2.21 (1.12-4.35)	**0.022**	3.67 (1.63-8.29)	**0.003**	3.40 (1.36-8.48)	**0.009**
Heart failure before iBAV	3.36 (1.78-6.34)	**<0.001**	5.32 (2.33-12.1)	**<0.001**	2.96 (1.20-7.29)	**0.018**
Duct dependent before iBAV	3.32 (1.76-6.25)	**<0.001**	5.61 (2.24-13.0)	**<0.001**	2.62 (1.06-6.45)	**0.037**
Ejection fraction before iBAV (%)	0.97 (0.95-0.98)	**<0.001**	0.96 (0.94-0.97)	**<0.001**	0.96 (0.94-0.98)	**<0.001**
LVIDd before iBAV (per 1-mm increase)	0.96 (0.89-1.04)	0.307	0.96 (0.86-1.06)	0.380	0.90 (0.80-1.01)	0.068
Moderate or severe aortic regurgitation after iBAV	1.67 (0.77-3.63)	0.195	0.57 (0.14-2.44)	0.452	2.48 (0.89-6.91)	0.082
Peak-to-peak gradient after iBAV (per 10-mmHg increase)	1.41 (1.08-1.84)	**0.010**	1.49 (1.08-2.06)	**0.014**	0.88 (0.56-1.38)	0.582
Mean echo gradient after iBAV (per 10-mmHg increase)	1.37 (1.05-1.77)	**0.017**	1.55 (1.13-2.14)	**0.007**	0.86 (0.57-1.29)	0.456
Use of rapid right ventricular pacing	0.39 (0.21-0.76)	**0.004**	0.14 (0.05-0.40)	**<0.001**	0.56 (0.22-1.44)	0.229
Multivariate analyses						
Unicommissural valve	1.73 (0.87-3.45)	0.119	2.92 (1.27-6.68)	**0.011**	3.69 (1.42-9.55)	**0.007**
Peak-to-peak gradient after iBAV (per 10-mmHg increase)	1.36 (1.02-1.79)	**0.037**	1.45 (1.02-2.06)	**0.039**	0.85 (0.54-1.33)	0.473
Age 0-1 month	2.10 (1.07-4.12)	**0.032**	2.19 (0.90-5.36)	0.086		
Moderate or severe aortic regurgitation after iBAV	1.88 (0.86-4.14)	0.116	0.58 (0.14-2.49)	0.465	3.31 (1.13-9.65)	**0.029**

Univariate and multivariate Cox regression analyses of periprocedural factors affecting hazard ratio for: 1) any reintervention (AVR, SAV, or BAV); 2) BAV reintervention; and 3) AVR. Age criterion not included in the multivariate AVR model due to sample size considerations. The **bold***P* values indicate significance at the 0.05 level.

AoV = aortic valve; AVR = aortic valve replacement; BAV = balloon aortic valvotomy; iBAV = index balloon aortic valvotomy; CI = confidence interval; HR = hazard ratio; LVIDd = left ventricular internal diameter end-diastole; SAV = surgical aortic valvotomy.

## Discussion

The current study examines children <1 year of age with valvar AS who were treated with BAV in the current era. Our main findings were that: 1) BAV can be performed safely in neonates and infants; 2) BAV effectively reduced aortic valve gradient; 3) the burden of AI increases steadily over time; 4) in the full cohort, freedom from AVR was 86% (79%-93%) and freedom from reintervention was 69% (78%-60%) at 10 years; and 5) in the subgroup of neonates, freedom from AVR was 79% (90%-68%) and freedom from reintervention was 56% (69%-42%) at 10 years.

### BAV is a safe initial treatment option for AS in infants and neonates

Treatment options for congenital AS remain contentious in the youngest of children. In our cohort, we report 3 (2%) deaths during follow-up, all occurring after AVR, but only 1 death occurred in the immediate postoperative period. None of the deceased children had severe AI promoting early valve replacement. Mortality rates in comparable cohorts range from 2.5 to 9% after SAV and 1.5 to 11% after BAV.^11^^,^^14^ The largest published cohort to date (647 neonates and infants) found a 10-year survival of 90.6% after BAV and 84.9% after SAV,^7^ and a recently published series of neonates reported a survival of 93.7% at 10 years after BAV.^17^ Taken together, our results are on par with published literature, which report no significant difference in mortality between primary interventional and surgical approaches.

The most common procedural complication was femoral artery thrombus requiring anticoagulation, occurring at a relatively high frequency of 28%. Many children regained pulsatile arterial flow during treatment with low-molecular-weight heparin, but the exact numbers are missing from our database. In comparison, Auld et al^8^ reported an incidence of 11% arterial thrombosis. The gap might be due to differences in weights, but also in the definition and reporting of complications. We had no complications when using the carotid artery for access. Although the numbers are small, this can be a reasonable option in smaller children with a higher risk of a femoral artery compromise.^18^

A time-dependent loss of valve competency has been reported after initial treatment with both BAV and SAV,^8^^,^^10^^,^^12^ constituting an important aspect of long-term management.^19^ In our cohort, moderate or severe AI developed acutely from none to 14% at the index BAV, with a further increase to 45% at the latest follow-up. Regurgitation, alone or as a mixed lesion, was the leading cause of both SAV reintervention and AVR. Published rates of moderate to severe AI range from 10 to 23% after BAV^6^^,^^12^^,^^14^^,^^17^ and 1 to 13% after SAV.^12^^,^^14^ Few studies report AI at the final follow-up, but Herrmann et al^12^ had an increase from 24% to 37% after BAV and 2 to 13% after SAV. Of note, that study had a mean age of 8 years in the SAV group and 6.5 years in the BAV group. Differences in ages and weights between study cohorts make outcome comparisons challenging. While the studies indicate an increased prevalence of moderate to severe AI after BAV, this is not reflected by an increased rate of AVR in the BAV populations.

### Freedom from AVR and reintervention

Most current studies comparing SAV and BAV report similar 10-year freedom of AVR at 79 to 87% after BAV and 80 to 90% after SAV.^7^^,^^9^ One exception is Vergant et al^13^ who reported fewer operations and AVRs after surgical valve reconstruction. Impressively, the subgroup of neonates where a trileaflet valve was achieved had a 10-year freedom from AVR at 95%. However, a trileaflet valve could only be achieved in 21 of 52 babies, leaving freedom from AVR at 79% for the full surgical cohort. We report a 10-year freedom from AVR at 87%, which compares favorably to both published interventional and surgical results. Of note, the subgroup of event-free survivors had an increase in moderate or severe AI from 10 to 40% during the study period (median: 7.1 years), anticipating a further increase in AVR in the future.

With similar rates of mortality and AVR, reintervention rates are commonly used to evaluate the safety and efficacy of initial management strategies. We report a 69% 10-year freedom from any form of reintervention. Siddiqui et al^11^ were 1 of the first to report better outcomes after an initial surgical approach, with a 5-year freedom from reintervention at 65% after SAV and 27% after BAV. Their study had only 37 infants in the BAV group, which may have contributed to the suboptimal results, and their SAV reintervention rate was similar to what can be observed after BAV. Two recent meta-analyses performed by Hill et al in 2016^9^ and Saung et al in 2019^10^ observed higher reintervention rates after BAV, while the multicenter study by McCrindle,^5^ the UK registry study by Dorobantu et al,^7^ and recent publications by Ivanov et al^21^ and Auld et al^14^ report similar freedom from reintervention, ranging from 40 to 70%. The studies differ in length of follow-up, and the indications for when to reintervene were not standardized, probably accounting for some of these differences.

Since what composes a reintervention is not uniformly defined in the literature, we argue that the general reintervention rate has a limited value in comparing techniques. In our population, 60% of first reinterventions were repeat BAVs, 28% were AVRs, and only 12% were SAVs. This reflects a practice at the index procedure to avoid creating regurgitation, and accepting a residual gradient, to improve overall hemodynamics, anticipating the requirement of later more aggressive percutaneous treatment.

### Predictors of AVR and reintervention

The strongest predictor of AVR in the current study was having a unicommissural aortic valve. Further, 22 of the 27 children with a unicommissural valve required intervention in the neonatal period, reflecting that children with the most anatomically abnormal valves required intervention at an earlier age and had worse outcome to interventional treatment. This is in line with the findings of Maskatia et al^20^ and Auld et al,^8^ while Ivanov et al^21^ found no association between valve morphology and the need for an AVR.

Neonates had an increased risk of reintervention in both univariate and multivariate analyses, which is supported by most published literature.^6^^,^^7^^,^^11^^,^^19^^,^^21^ However, factors predicting the need for reintervention (eg, age at the index BAV, fraction of children with heart failure and ductal dependency before BAV, lower Doppler gradients before BAV and lower LV ejection fractions before and after BAV) were similar in the total population and in the neonatal subgroup. We therefore argue that the underlying anatomy and resulting hemodynamic consequences are more important for prognosis than the child’s age alone.

### Study Limitations

Exclusion criteria were set to address children where the main problem was valvar AS, excluding more complex cardiac lesions. This might have reduced generalizability. The cohort size and number of events are limited, giving limited statistical power in predictor analyses. Missing a surgical comparison group excludes the study from reporting on outcome differences. A longer follow-up time may have allowed a better understanding of the long-term freedom from AVR.

## Conclusions

BAV is a safe and effective initial management strategy for neonatal and infant AS. Progressive AI constitutes an important aspect of long-term follow-up. Outcomes are competitive with contemporary published results after surgical valve repair in relation to mortality, gradient relief, long-term AVR, and reintervention rates. In the absence of significant AI, surgery can be reserved for those with gradients resistant to valve dilation.

PERSPECTIVES **COMPETENCY IN MEDICAL KNOWLEDGE 1:** Contemporary treatment of congenital aortic stenosis is contentious in neonates and infants, with both balloon valvotomy and surgical valve repair being used with good results.

**COMPETENCY IN PATIENT CARE AND PROCEDURAL SKILLS:** In the current interventional cohort, outcomes were found competitive with published results after aortic valve repair in relation to mortality, gradient relief, long-term valve replacement, and reintervention rates.

**COMPETENCY IN MEDICAL KNOWLEDGE 2:** In the absence of significant aortic regurgitation, surgery can be reserved for those with gradients resistant to valve dilation.

## Funding Support and Author Disclosures

The authors have reported that they have no relationships relevant to the contents of this paper to disclose.
