# Supplementary material for: Current-Era Outcomes of Balloon Aortic Valvotomy in Neonates and Infants
Source: JACC Adv. 2022 Mar 16;1(1):100004. doi: 10.1016/j.jacadv.2022.100004 (PMC11198319; doi:10.1016/j.jacadv.2022.100004)
Supplement: Supplemental Figures 1 and 2, and Supplemental Table 1 [file mmc1.docx]

Included patients

(n = 139)

Total population

(n = 157)

Borderline left ventricle

(n = 10)

Shone complex

(n = 4)

Poly-valvar disease

(n = 3)

Primary pulmonary hypertension

(n = 1)

HTx

(n=2)

Ross

(n=2)

Deceased

(n=1)

Hybrid

(n=8)

Nw1

(n=1)

Bi-Ventricular repair

(n=4)

Deceased

(n=2)

Conversion to Nw1

(n=1)

HTx

(n=2)

Deceased

(n=1)

MV Dysplasia

(n=2)

PA/IVS

(n=1)

Deceased

(n=1)

Deceased

(n=1)

Deceased

(n=1)

Deceased (n=1)

Deceased (n=3)

**Supplemental Figure 1. Excluded patients.**

Flowchart describing excluded patients. HTx: heart transplantation, MV dysplasia: severe mitral valve dysplasia requiring valve replacement, Nw1: Norwood operation stage one, PA/IVS: Pulmonary atresia with intact ventricular septum.

An average of 8.7 index BAVs (range 2 to 18) were performed per year in the study period from January 2004 to July 2019. BAV: balloon aortic valvotomy.

**Supplemental Figure 2. Number of index BAVs performed per year**

| **Supplemental Table 1. Short and long-term efficacy of index BAV** | | | | | | | | |
| --- | --- | --- | --- | --- | --- | --- | --- | --- |
|  | Before iBAV | 95% CI | After iBAV | 95% CI | p^*^ | Final visit | 95% CI | p^#^ |
| *Echocardiography* |  |  |  |  |  |  |  |  |
| AoV peak gradient - mmHg, mean (SD) | 96 (30) | 91 to 101 | 49 (21) | 45 to 52 | **<0.001** | 46 (25) | 42 to 51 | 0.432 |
| AoV mean gradient - mmHg, mean (SD) | 53 (16) | 51 to 56 | 26 (12) | 24 to 28 | **<0.001** | 25 (14) | 23 to 28 | 0.843 |
| AoV stenosis, number (%) |  |  |  |  | **<0.001** |  |  | 0.688 |
| non or mild (mean gradient < 20mmHg) | 4 (3%) |  | 51 (39%) |  |  | 52 (41) |  |  |
| moderate (mean gradient 20 - 40 mmHg) | 18 (13%) |  | 61 (47%) |  |  | 57 (45) |  |  |
| severe (mean gradient > 40mmHg) | 115 (84%) |  | 19 (15%) |  |  | 19 (15) |  |  |
| AoV regurgitation, number (%) |  |  |  |  | **<0.001** |  |  | **<0.001** |
| Non or trivial | 137 (99%) |  | 59 (44%) |  |  | 31 (23%) |  |  |
| mild | 2 (1%) |  | 56 (42%) |  |  | 43 (32%) |  |  |
| moderate | 0 (0%) |  | 13 (10%) |  |  | 34 (25%) |  |  |
| severe | 0 (0%) |  | 5 (4%) |  |  | 27 (20%) |  |  |
| Ejection fraction - %, mean (SD) | 67 (18) | 63 to 70 | 69 (15) | 67 to 72 | **<0.001** | 71 (8) | 70 to 72 | 0.346 |
| *Catheterization* |  |  |  |  |  |  |  |  |
| LV systolic pressure - mmHg, mean (SD) | 110 (22) | 107 to 114 | 84 (18) | 81 to 87 | **<0.001** |  |  |  |
| LV end-diastolic pressure - mmHg, mean (SD) | 13 (7) | 12 to 15 | 12 (6) | 11 to 13 | **0.045** |  |  |  |
| AAo systolic pressure - mmHG, mean (SD) | 59 (15) | 57 to 61 | 66 (16) | 63 to 68 | **<0.001** |  |  |  |
| AAo diastolic pressure - mmHG, mean (SD) | 36 (9) | 34 to 37 | 36 (9) | 35 to 38 | 0.289 |  |  |  |
| AAo mean pressure - mmHG, mean (SD) | 46 (11) | 44 to 48 | 49 (11) | 47 to 51 | **<0.001** |  |  |  |
| AoV peak to peak gradient - mmHg, mean (SD) | 52 (21) | 48 to 54 | 18 (11) | 16 to 21 | **<0.001** |  |  |  |
| Statistical differences were examined using paired samples Student t-test or Wilcoxon signed rank test as appropriate. p^*^ = tests between: before and after index BAV, p^#^ = tests between: after index BAV and final visit. The before and after echocardiograms were performed within days of the iBAV. The final visit was median 7.1 years after iBAV. Abbv: AAo Ascending aorta, AoV: aortic valve, CI: confidence interval, iBAV: index balloon aortic valvulotomy, LV: left ventricle SD: standard deviation | | | | | | | | |
